# Transfer RNA-derived small RNAs and their potential roles in the therapeutic heterogeneity of sacubitril/valsartan in heart failure patients after acute myocardial infarction

**DOI:** 10.3389/fcvm.2022.961700

**Published:** 2022-09-29

**Authors:** Jia Su, Ji Cheng, Yingchu Hu, Qinglin Yu, Zhenwei Li, Jiyi Li, Nan Zheng, Zhaoxia Zhang, Jin Yang, Xiaojing Li, Zeqin Zhang, Yong Wang, Keqi Zhu, Weiping Du, Xiaomin Chen

**Affiliations:** ^1^Department of Cardiology, Ningbo No. 1 Hospital, Ningbo, Zhejiang, China; ^2^Key Laboratory of Precision Medicine for Atherosclerotic Diseases of Zhejiang Province, Ningbo, Zhejiang, China; ^3^Department of Emergency, HwaMei Hospital, University of Chinese Academy of Sciences, Ningbo, Zhejiang, China; ^4^Department of Traditional Chinese Internal Medicine, Ningbo No. 1 Hospital, Ningbo, Zhejiang, China; ^5^Department of Cardiology, Yuyao People’s Hospital of Zhejiang Province, Yuyao, Zhejiang, China; ^6^Department of Cardiology, HwaMei Hospital, University of Chinese Academy of Sciences, Ningbo, Zhejiang, China; ^7^Department of Geriatrics, Ningbo No. 1 Hospital, Ningbo, Zhejiang, China

**Keywords:** tsRNA, expression profile, sacubitril/valsartan, lipid and atherosclerosis signal pathway, heart failure

## Abstract

**Background:**

It has been reported that sacubitril/valsartan can improve cardiac function in acute myocardial infarction (AMI) patients complicated by heart failure (HF). However, a number of patients cannot be treated successfully; this phenomenon is called sacubitril/valsartan resistance (SVR), and the mechanisms remain unclear.

**Methods:**

In our present research, the expression profiles of transfer RNA (tRNA)-derived small RNAs (tsRNAs) in SVR along with no sacubitril/valsartan resistance (NSVR) patients were determined by RNA sequencing. Through bioinformatics, quantitative real-time PCR (qRT-PCR), and cell-based experiments, we identified SVR-related tsRNAs and confirmed their diagnostic value, predicted their targeted genes, and explored the enriched signal pathways as well as regulatory roles of tsRNAs in SVR.

**Results:**

Our research indicated that 36 tsRNAs were upregulated and that 21 tsRNAs were downregulated in SVR. Among these tsRNAs, the expression of tRF-59:76-Tyr-GTA-2-M3 and tRF-60:76-Val-AAC-1-M5 was upregulated, while the expression of tRF-1:29-Gly-GCC-1 was downregulated in the group of SVR. Receiver operating characteristic (ROC) curve analysis demonstrated that these three tsRNAs were potential biomarkers of the therapeutic heterogeneity of sacubitril/valsartan. Moreover, tRF-60:76-Val-AAC-1-M5 might target *Tnfrsf10b* and *Bcl2l1* to influence the observed therapeutic heterogeneity through the lipid and atherosclerosis signaling pathways.

**Conclusion:**

Hence, tsRNA might play a vital role in SVR. These discoveries provide new insights for the mechanistic investigation of responsiveness to sacubitril/valsartan.

## Introduction

Acute myocardial infarction (AMI) is one of the most critical cardiovascular diseases in humans, and heart failure (HF) is the most common complication after AMI. Evidence has shown that the probability of HF within 30 days to 6.7 years after the onset of AMI is as high as 13.1–37.5% (1). AMI combined with HF often indicates a poor prognosis and is associated with mortality 20.9 times higher than that of patients without HF (2). Therefore, HF has become an important factor affecting the long-term survival of myocardial infarction patients and their quality of life.

The pathogenesis of HF after AMI is related to the activation of the neuroendocrine system, ventricular remodeling, and inflammatory factors (3, 4). In the early stage of myocardial infarction, a large number of cardiomyocytes undergo necrosis and apoptosis, which reduces myocardial contractility and ventricular output, resulting in heart pump failure and compensatory activation of the renin-angiotensin-aldosterone system (RAAS) as well as natriuretic peptide system. Sacubitril/valsartan, which was the first dual inhibitor of enkephalinase and angiotensin receptor to be developed, can simultaneously regulate the natriuretic peptide system and RAAS and plays a dual cardioprotective role (5). The multicenter, randomized, double-blind, phase III PARADISE-MI study revealed that sacubitril/valsartan treatment could delay or reverse ventricular remodeling and improve cardiac function in patients with AMI complicated by HF (6). However, there is variation in the response to sacubitril/valsartan treatment, which may be related to the clinical characteristics and genetic factors of patients. One study showed that after treatment with sacubitril/valsartan, 31% of patients showed no change in the left ventricular ejection fraction (LVEF), and 19% showed a decrease in LVEF, which may be related to a lower basic LVEF value, non-ischemic cardiomyopathy, the lack of an implantable cardioverter defibrillator and a shorter history of HF (7). We refer to this phenomenon of the therapeutic heterogeneity of sacubitril/valsartan as sacubitril/valsartan resistance (SVR). A sum of clinical research works have discovered that SVR may be interrelated to the medication dose, troponin, sex, race, left ventricular end-systolic volume, etiology, and other factors (8–12). However, there is a lack of in-depth research concerning the specific pharmacodynamic mechanism of SVR at present.

Transfer RNA (tRNA) is a kind of RNA that is widespread in the human body, accounting for 4–10% of total cellular RNA. More than 500 tRNAs have been identified in the human body to date (13), and approximately half of them have been proven to come from actively expressed genes (14). When cells are exposed to conditions such as hypoxia, oxidative stress, starvation and high temperature, precursor and mature tRNAs can be cleaved by corresponding nucleases to form tRNA-derived small RNAs (tsRNAs) with specific functions (15–17). tsRNA retains the diversity of tRNA functions and shows the characteristics of high expression, fixed conservation, and good stability (18). It can play a role as an epigenetic regulator of mRNA stability, protein translation, and gene expression (16). tsRNA can be considered not only a therapeutic target but also a biomarker that may be used in diagnosis and prognosis assessment (19), and one study has shown that tRF-Gly-GCC can promote the regulation of cell adhesion, proliferation, migration as well as phenotypic transformation in Human umbilical vein endothelial cells (HUVECs) and vascular smooth muscle cells (VSMCs) by inhibiting the expression of MERVL-related genes (20). tsRNA has now been confirmed to produce a marked effect on cardiovascular diseases (21, 22).

Therefore, this study aimed to screen the differential tsRNA expression profiles of patients with SVR or with no sacubitril/valsartan resistance (NSVR) and to explore the possible underlying molecular mechanism to achieve greater prognostic benefits and realize individualized treatment.

## Materials and methods

### Study population

From October 2019 to December 2021, patients who were diagnosed with ST-elevation AMI with a reduced ejection fraction in HF were selected from Ningbo No. 1 Hospital, on the east coast of China. They all came from the Han population, had undergone complete revascularization, and had received the maximum tolerated dose of sacubitril/valsartan for at least 6 months. According to the baseline LVEF and the changes in LVEF after sacubitril/valsartan administration, the patients in this study were divided into NSVR group and SVR group: (1) a cardiac function improvement group, referred to as the NSVR group, showing an LVEF >45 and ≥10% higher than baseline at the last review; and (2) a group with poor improvement of cardiac function, referred to as the SVR group, showing an LVEF ≤40 or <10% higher than baseline at the last review.

The clinical data collected included basic clinical information (age, sex, height, weight, BMI, systolic blood pressure, smoking and alcohol history, coexisting diseases, and so on), blood laboratory test indices (e.g., blood routine, CRP, hepatic and renal function, blood glucose and lipid, coagulation function, aTnI, and NT-proBNP results), cardiac ultrasound indexes before and after treatment with sacubitril/valsartan (LVEF, left ventricular diastolic diameter, and left ventricle systolic diameter), coronary artery disease characteristics, and the clinical medication regime (medication strategy, dosage, and time).

This study was approved by the ethics committee of Ningbo No. 1 Hospital, and every person Included signed a written informed consent form before the investigation. The whole research was implemented in line with the principles of the Helsinki Declaration.

### Inclusion and exclusion criteria

The studied patients were included in the light of the followings: (1) age greater than 18 years and less than 75 years; (2) meeting the diagnostic criteria for ST-elevate AMI (23); (3) having undergone complete revascularization; (4) LVEF ≤45%; (5) a Killip classification (24) of Class II–IV; and (6) having been continuously and regularly treated for 6 months or more.

If any of the following criteria were met, the patient was excluded: (1) contraindications for sacubitril/valsartan (including systolic blood pressure <95 mmHg, potassium >5.4 mmol/L, glomerular filtration rate <30 ml/min/1.73 m^2^); (2) cardiac insufficiency due to other heart diseases (e.g., valvular heart disease, dilated cardiomyopathy, and hypertensive cardiac insufficiency); (3) pregnancy or lactation in women; or (4) incomplete information.

### RNA extraction and transfer RNA-derived small RNAs sequencing

From 22 subjects with SVR and equal numbers of NSVR, the peripheral blood samples were collected after 6 months of sacubitril/valsartan treatment. Ten of these patients were used for the systemic analysis of tsRNA expression profiles (these 5 SVRs and 5 NSVRs were well-matched), and the remaining samples (17 SVR along with 17 NSVR) were arranged for validation.

After PBMC isolation, total RNA in samples were obtained by using TRIzol reagent, and the purity and concentration of them were determined with the instrument of NanoDrop ND-1000. If the OD was in the range of 1.8–2.0, the RNA was included in the follow-up experiment. According to rtStar tRF and tiRNA Pretreatment Kit protocols (Arraystar, USA), the total RNA samples were pretreated to remove RNA modifications, which would interfere with the library construction of small RNA-seq.

Then, the samples were sequentially ligated to 3′ and 5′ small RNA adapters. After adding Illumina’s proprietary RT primers and amplification primers, cDNA was synthesized and amplified. Next, the PCR-amplified fragments (134–160 bp PCR amplified fragments corresponding to 14–40 nt small RNA size range) were extracted and purified from the PAGE gel. And with an Agilent 2100 Bioanalyzer, the integrated libraries were quantified, denatured and diluted. Lastly, according to the manufacturer’s instructions, the diluted libraries were loaded onto a reagent cartridge and forwarded for sequencing using NextSeq 500/550 High Output v2 Kits (FC-404-2004, Illumina, USA).

### Data analysis

Base detection and image analysis were operated with the Solexa Pipeline, and the original data were subjected to quality inspection with FastQC software. The trimmed valid data were compared with pre-tRNA and mature tRNA sequences by using Bowtie software. These trimmed reads were aligned to mature-tRNA and pre-tRNA sequences from GtRNAdb^1^ and tRNAscan-SE.^2^ The exactly matched reads were thought as tsRNAs. In view of comparative statistical analysis, the tsRNA expression profiles of the SVR and NSVR groups were finally obtained.

The differentially expressed tsRNAs were analyzed with R package edgeR by GEO (Gene Expression Omnibus^3^). The calculated *P*-values and fold changes (FCs) were used to compare the tsRNAs of the two groups. A FCs more than 1.5 and *P* value less than 0.05 were considered significant. Then, the differentially expressed tsRNAs were represented *via* volcano map, heatmap, and scatter map.

### Validation by quantitative real-time PCR

Five treatment-related tsRNAs (tRF-1:28-Gly-GCC-1, tRF-1:29-Glu-CTC-1-M2, tRF-1:29-Gly-GCC-1, tRF-59:76-Tyr-GTA-2-M3, and tRF-60:76-Val-AAC-1-M5) showing relatively high FC values were selected for cross-validation through quantitative real-time PCR (qRT-PCR) in triplicate.

First, applying the rtStar™ tRF and tiRNA First-Strand cDNA Synthesis Kit (Arraystar), total RNA was reverse-transcribed into cDNA according to the manufacturer’s protocol. Then, qRT-PCR amplification was carried out through an ABI 7500 qRT-PCR System (Applied Biosystems, Foster City, CA, USA) and 2 × PCR master mix (Arraystar). The conditions of PCR amplification were as followings: (1) incubation (95°C, 10 min), (2) 40 PCR cycles (95°C, 10 s; 60°C, 60 s for fluorescence collection), and (3) after the PCR amplification reaction, the melting curve was established on the basis of the following program: 95°C, 10 s; 60°C, 60 s; 95°C, 15 s. The levels of relative tsRNA expression were reckoned by the 2^–Δ^ ^Δ^ *^Ct^* method and were normalized to U6 as a housekeeping gene.

The target mRNAs of selected tsRNAs were confirmed *via* qRT-PCR too. The protocol was similar to that described above, including cDNA synthesis with the PrimeScript™ RT Reagent Kit and gDNA Eraser (TaKaRa Bio, Kusatsu, Japan), template cDNA dilution and real-time PCR. Here, GAPDH was set as normalization.

The software of Premier 8.0 was applied to primers design (Table 1) for tsRNA and mRNA in the qRT-PCR assays.

**TABLE 1:** Specific primers for tsRNA and mRNA in qRT-PCR.

Gene name	Primer sequence	Product length (bp)
U6	F:5′GCTTCGGCAGCACATATACTAAAAT3′	89
	R:5′CGCTTCACGAATTTGCGTGTCAT3′	
tRF-60-76-Val-AAC-1-M5	F:5′CTACAGTCCGACGATCACCG3’	42
	R:5′TTCCGATCTTGGTGTTTCCG3′	
tRF-59-76-Tyr-GTA-2-M3	F:5′CTACAGTCCGACGATCTTCCG3′	47
	R:5′GCTCTTCCGATCTTGGTCCTT3′	
tRF-1-28-Gly-GCC-1	F:5′TACAGTCCGACGATCGCATG3′	50
	R:5′CCGATCTGAGAATTCTACCACTGA3′	
tRF-1-29-Gly-GCC-1	F:5′GATCGCATGGGTGGTTCAGT3′	53
	R:5′GACGTGTGCTCTTCCGATCTC3′	
tRF-1-29-Glu-CTC-1-M2	F:5′ATCTCCCTGGTGGTCTAGTGGT3′	50
	R:5′CGTGTGCTCTTCCGATCTCC3′	
GAPDH (HUMAN)	F:5′GGGAAACTGTGGCGTGAT3′	299
	R:5′GAGTGGGTGTCGCTGTTGA3′	
CAMK2B	F:CAGATGGAGTCAAGCCCCAG	109
	R:TGGATGACGGTGGTTTGAGG	
VAV3	F:AGTGGTGTTTTACCACTCTGC	134
	R:TCCATTGGTCCGTTTCTCTGG	
PLCB3	F:CATCAGGGACACACGGACAG	200
	R:GCTCCTCAGACCAGACCTTG	
NFATC3	F:CTGACTTGGAACACCAGCCA	112
	R:TCTCCCAATTATCTCGTTCACCTC	
TNFRSF10B	F:CCCTGTTCTCTCTCAGGCATC	175
	R: CAGGTCGTTGTGAGCTTCTGT	
BCL2L1	F:TTCTGGGCTCCCAGCCT	199
	R:TCTGAAGGGAGAGAAAGAGATTCA	

### Target mRNA prediction of treatment-related transfer RNA-derived small RNAs

Transfer RNA-derived small RNAs contain many seed sequences that may correspond to the cross-linked central regions of their target mRNAs. As tsRNAs show an miRNA-like function and can silence the expression of their target mRNAs, our group searched two algorithms to predict target mRNAs of treatment-related tsRNAs: tRFTar^4^ and tsRFun^5^ (25, 26). The genes, which were predicted by both algorithms at the same time, were considered significant.

### Bioinformatic analysis

The target genes corresponding to the differentially expressed tsRNAs were analyzed by Gene Ontology (GO) annotation as well as Kyoto Encyclopedia of Genes and Genomes (KEGG) annotation.

The GO database is used for the classification of gene function and is the most comprehensive and widely used knowledge base focused on gene function. The functions indicated by the GO analysis of genes are divided into three major categories: cellular component (CC), molecular function (MF), and biological process (BP). Each GO entry corresponds to a *P*-value indicating significance, where the smaller the *P*-value is, the stronger the relationship between the GO entry and the corresponding differentially expressed gene.

Kyoto Encyclopedia of Genes and Genomes is a database for systematically analyzing the metabolic pathways of gene products in cells and the functions of these gene products, which is helpful for understanding the higher-level systemic functions of cells and organisms, such as metabolic processes in cells, human diseases and biological functions. The smaller the *P*-value of each KEGG entry, the greater the relationship between the signal pathway corresponding to the KEGG entry and the differentially expressed gene.

### Cell culture and transfection

Human umbilical vein endothelial cells obtained commercially from Haixing Biosciences (Suzhou, China) were cultured in sterile and thermostatic cell incubators with RPMI 1640 medium (Thermo Fisher Scientific, USA) supplemented with 10% fetal bovine serum (Thermo Fisher Scientific, USA). The cells were incubated in 5% carbon dioxide with saturated humidity at 37°C. During the exponential phase of growth, HUVECs were seeded into six-well plates at a density of 2 × 10^6^ cells/well for transfection. The tRF-60:76-Val-AAC-1-M5 mimic (ACCGGGCGGAAACACCA) and the negative control (NC; UUUGUACUACACAAAAGUACUG) were obtained from Aksomics (Shanghai, China). According to the manufacturer’s instructions, the transfection of the mimic and NC was operated through Lipofectamine 3000 (Invitrogen, USA) at the concentration of 100 nmol. All experiments were performed in triplicate. And 48 h later, the transfected cells were harvested for RNA isolation. The relative mRNA levels of hsa05417 related genes were tested by qPCR. The specific primers are summarized in Table 1, and the relevant protocols were the same those as described above.

### Statistic analysis

The statistic analysis was carried out with SPSS 22.0 software. The measurement data conforming to the normal distribution were expressed as the mean ± SD, and the comparisons between the two groups were performed *via t*-test. If the measurement data did not conform to a normal distribution, the data were analyzed with a non-parametric test. Count data were expressed as frequencies and percentages. The χ^2^ test or Fisher’s exact probability method was applied for comparisons between these groups. The diagnostic value of tsRNA was assessed *via* receiver operating characteristic (ROC) curve analysis. GraphPad Prism 8.0 software was adopted to assemble the figure panels.

## Results

### Characteristics of the studied patients

A sum of 44 subjects (22 SVR and 22 NSVR) were included in our research. Ten of them were used for tsRNA expression profile analysis (5 SVR and 5 NSVR patients), and the other samples (17 SVR and 17 NSVR) were used for validation.

The clinical characteristics of these 10 patients (5 SVR and 5 NSVR) are presented in Table 2. Except for the ARNI effect, the clinical baselines were identically matched, and no remarkable differences were observed between these two groups. The characteristics of the validation cohort are listed in Table 3. There was no obvious difference between the SVR and NSVR groups in terms of the basic clinical data, cardiac structure, or medication used except for the ARNI effect.

**TABLE 2:** The clinical characteristics of the SVR and NSVR patients included in tsRNA expression profile analysis.

	SVR (*n* = 5)	NSVR (*n* = 5)	*P*-value
Age (year)	70.4 ± 7.20	67.8 ± 14.24	0.725
Male, *n* (%)	3 (60)	5 (100)	0.444
BMI (kg/m^2^)	25.45 ± 6.25	24.18 ± 0.85	0.676
Hypertension, *n* (%)	4 (80)	4 (80)	–
Diabetes, *n* (%)	3 (60)	1 (20)	0.524
Hyperlipidemia, *n* (%)	4 (80)	2 (40)	0.524
Smoking history, *n* (%)	2 (40)	4 (80)	0.524
Drinking history, *n* (%)	1 (20)	1 (20)	–
aTnI (μg/L)	20.65 ± 29.28	39.54 ± 45.72	0.459
TC (mmol/L)	5.02 ± 1.31	4.61 ± 1.65	0.676
TG (mmol/L)	1.55 ± 0.35	1.19 ± 0.24	0.095
HDL-C (mmol/L)	1.06 ± 0.27	1.01 ± 0.28	0.746
LDL-C (mmol/L)	3.52 ± 0.91	3.32 ± 1.21	0.775
APoE (mg/L)	48.22 ± 9.27	40.56 ± 10.33	0.252
ALT (U/L)	35.6 ± 19.69	54.8 ± 54.36	0.479
AST (U/L)	106 ± 88.39	326.6 ± 490.43	0.375
TBIL (μmol/L)	19.5 ± 13.92	13.34 ± 2.62	0.359
Albumin (g/L)	36.18 ± 1.64	37.88 ± 1.05	0.087
BUN (mmol/L)	7.19 ± 3.73	5.77 ± 0.84	0.449
CREA (μmol/L)	82.4 ± 17.47	98.2 ± 20.39	0.225
UA (μmmol/L)	435.94 ± 138.42	442.48 ± 72.66	0.928
HbA1C (%)	6.62 ± 1.06	6.46 ± 0.94	0.807
CRP (mg/L)	2.11 (0.68–28.70)	3.81 ± 4.64	0.600
PLT (*10^9^/L)	202 ± 23.14	201.6 ± 62.34	0.990
MPV (fL)	11.3 ± 0.72	10.48 ± 0.72	0.110
PDW (fL)	14.22 ± 1.85	12.6 ± 1.92	0.211
**Before treatment of sacubitril/valsartan**			
LVEF (%)	39.2 ± 5.45	42.4 ± 1.95	0.251
Pro-BNP (pg/ml)	1,816.94 ± 2,304.4	2,854.1 ± 2,284.87	0.495
**After treatment of sacubitril/valsartan 6 months**			
LVEF (%)	54.4 ± 4.62	43 ± 1.58	0.001
Pro-BNP (pg/ml)	268.64 ± 336.2	2,154.94 ± 1,810.34	0.080

**TABLE 3:** The clinical characteristics of SVR and NSVR patients in the tsRNA validation cohort.

	Total (*n* = 34)	SVR (*n* = 17)	NSVR (*n* = 17)	*P*-value
Age (year)	64.48 ± 11.48	64.81 ± 12.03	66.03 ± 10.48	0.11
Male, *n* (%)	26 (76.5)	13 (76.5)	13 (76.5)	–
BMI (kg/m^2^)	23.38 ± 4.07	22.74 ± 4.69	24.20 ± 2.93	0.136
Smoking history, *n* (%)	19 (55.9)	9 (52.9)	10 (58.8)	0.730
Drinking history, *n* (%)	8 (23.5)	5 (29.4)	3 (17.6)	0.686
Hypertension, *n* (%)	22 (64.7)	11 (64.7)	11 (64.7)	–
Diabetes, *n* (%)	9 (26.5)	5 (29.4)	4 (23.5)	0.697
Hyperlipidemia, *n* (%)	22 (64.7)	12 (70.6)	10 (58.8)	0.720
Atrial fibrillation, *n* (%)	3 (8.8)	2 (11.8)	1 (5.9)	0.545
**Cardiac function classification**				
II	13 (38.2)	6 (35.3)	7 (41.2)	0.724
III	15 (44.1)	9 (52.9)	6 (35.3)	0.490
IV	6 (17.6)	4 (23.5)	2 (11.8)	0.656
Pro-BNP (pg/ml)	2,508.22 ± 3,328.71	2,608.59 ± 3,742.49	2,378.49 ± 2,742.82	0.867
aTnI (μg/L)	19.62 ± 26.68	21.95 ± 29.38	17.81 ± 24.54	0.459
TC (mmol/L)	4.16 ± 1.26	4.34 ± 1.20	3.93 ± 1.30	0.121
LDL-C (mmol/L)	2.78 ± 0.97	2.88 ± 0.92	2.67 ± 1.02	0.285
CREA (μmol/L)	86.56 ± 24.62	85.70 ± 25.71	87.66 ± 23.42	0.704
CRP (mg/L)	19.74 ± 34.80	21.06 ± 40.21	8.03 ± 26.63	0.438
**Ultrasonic data**				
LVEF (%)	41.79 ± 10.59	39.50 ± 10.01	42.76 ± 10.71	0.266
LVEDD (mm)	58.67 ± 7.91	58.96 ± 7.12	58.47 ± 6.01	0.085
LVESD (mm)	48.11 ± 9.24	47.56 ± 6.01	48.60 ± 9.78	0.147
**Coronary targeted vessel *n* (%)**				
LAD		12 (70.6)	10 (58.8)	0.473
LCX	5 (14.7)	2 (11.8)	3 (17.6)	0.628
RAD	7 (20.6)	3 (17.6)	4 (23.5)	0.571
**Meditation *n* (%)**				
Aspirin	34 (100)	17 (100)	17 (100)	–
Ticagrelor	21 (61.8)	12 (70.6)	9 (52.9)	0.480
Clopidogrel	13 (38.2)	7 (53.8)	6 (46.2)	0.724
Rivaroxaban	4 (11.8)	3 (17.6)	1 (5.9)	0.595
Dabigatran	2 (5.9)	1 (5.9)	1 (5.9)	−0.287
Statin	34 (100)	17 (100)	17 (100)	–
Ezetimibe	12 (35.3)	8 (47.1)	4 (23.5)	0.151
SGLT-2 inhibitor	6 (17.6)	2 (11.8)	4 (23.5)	0.368
Digoxin	2 (5.9)	0 (0)	2 (11.8)	0.466
Diuretic	19 (55.9)	8 (47.1)	11 (64.7)	0.490
Beta blocker	30 (88.2)	16 (94.1)	14 (82.4)	0.287
Calcium antagonist	2 (5.9)	1 (5.9)	1 (5.9)	–
Ivabradine	3 (8.8)	2 (11.8)	1 (5.9)	0.545

### Altered expression profiles of transfer RNA-derived small RNAs in sacubitril/valsartan resistance and no sacubitril/valsartan resistance patients

**FIGURE 1:**
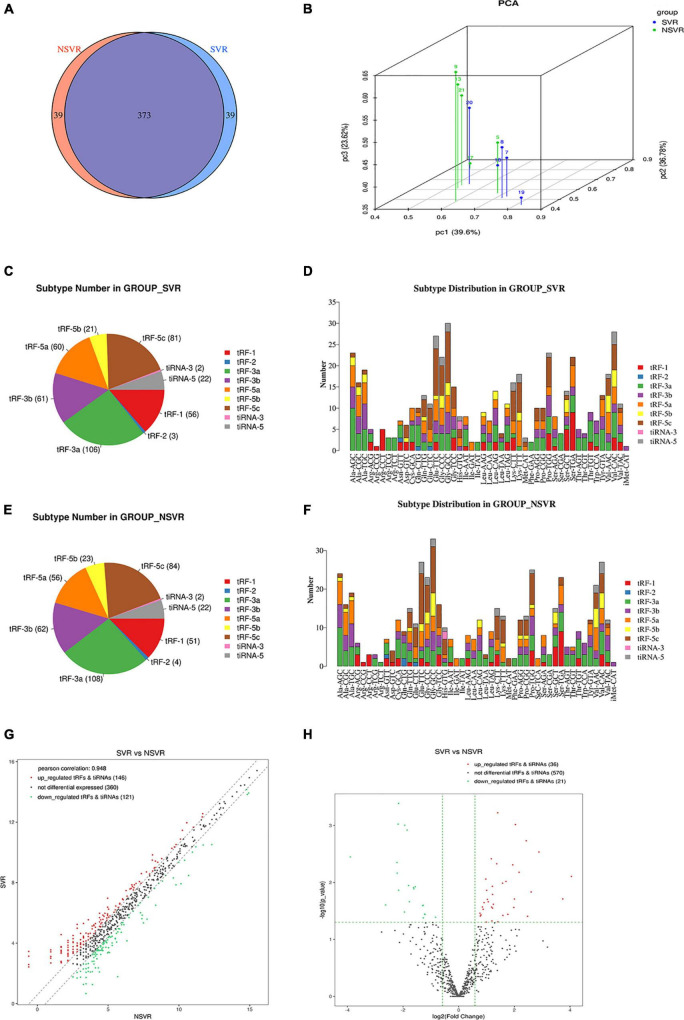
Altered expression profiles of tsRNAs in SVR and NSVR patients. **(A)** The Venn diagram shows the number of tRFs and tiRNAs expressed in SVR and NSVR patients. **(B)** Principal component analysis. The PCA was performed with tRF and tiRNA that have the ANOVA *P*-value ≤ 0.05 on CPM value and showed a distinguishable tRF and tiRNA expression profiling among the samples. The *X*, *Y*, and *Z* axis represents the three main factors which affected the expression level of the sample. The colored point represents the corresponding sample, and the location of it shows the main character of the sample. Space distance represents the similarity of data size. **(C)** The number of subtypes of tRF and tiRNA against tRNA isodecoders in SVRs. **(D)** The distribution of tRF and tiRNA subtypes in SVRs. **(E)** The number of subtypes of tRF and tiRNA against tRNA isodecoders in NSVRs. **(F)** The distribution of tRF and tiRNA subtypes in NSVRs. **(G)** Scatter plot between the two groups for tRF and tiRNA. **(H)** Volcano plot of tRFs and tiRNAs.

Transfer RNA-derived small RNAs sequencing (tsRNA-Seq) analysis (the data has been successfully deposited on the web of GEO^6^) was used to assess the differential expression of tsRNAs in SVR and NSVR patients. The reads quality score and statistical information are shown in Supplementary Tables 1, 2. The aligned percentage depends on multiple factors, including sample quality, library quality, and sequencing quality. Table 4 was listed top five tsRNAs with the highest expression fold changes. A total of 683 tsRNAs were sequenced in present research, after comparing alignments with GtRNAdb (see text footnote 1) and tRNAscan-SE (see text footnote 2), of which 152 were included in the tRF database (mintbase and tRFbd), and the remaining 531 were not found in the database (Figures 1A,B). Figures 1C,E show the distribution of the numbers of tsRNA subtypes with a group average threshold Log_2_(CPM) ≥ 20 sequenced in this study. The tsRNA subtypes were reclassified according to the differences in the amino acids transported by the source tRNA and anti-codons (Figures 1D,F). We also drew a scatter diagram (Figure 1G) and a volcano diagram (Figure 1H) according to the expression differences between the SVR and NSVR groups. In the scatter plot, in SVR group, 146 tsRNAs were upregulated, and 121 tsRNAs were downregulated. As show in the volcano plot, 36 tsRNAs were upregulated and 21 were downregulated in SVR group.

**TABLE 4:** Top five tsRNAs with the highest expression fold changes.

tRF_ID	log2FC	FC	*P*-value	*q*-value	Test_ Log_2_(CPM)	Control_ Log_2_(CPM)
tRF-30:43-Glu-CTC-1-M2	4.05	16.58	7.84e−03	3.07e−01	3.45	−0.61
tRF-55:76-Lys-TTT-3-M2	3.74	13.37	1.95e−02	4.01e−01	3.14	−0.61
tRF-56:77-Ile-AAT-1-M4	2.88	7.38	2.92e−03	2.29e−01	4.56	1.68
tRF-59:77-Ile-AAT-1-M4	2.60	6.08	1.47e−02	3.41e−01	3.55	0.95
tRF-+1:T19-Asp-GTC-M1	2.48	5.56	3.93e−02	4.74e−01	3.42	0.95

### Identification of sacubitril/valsartan resistance-related transfer RNA-derived small RNAs and qPCR confirmation

**FIGURE 2:**
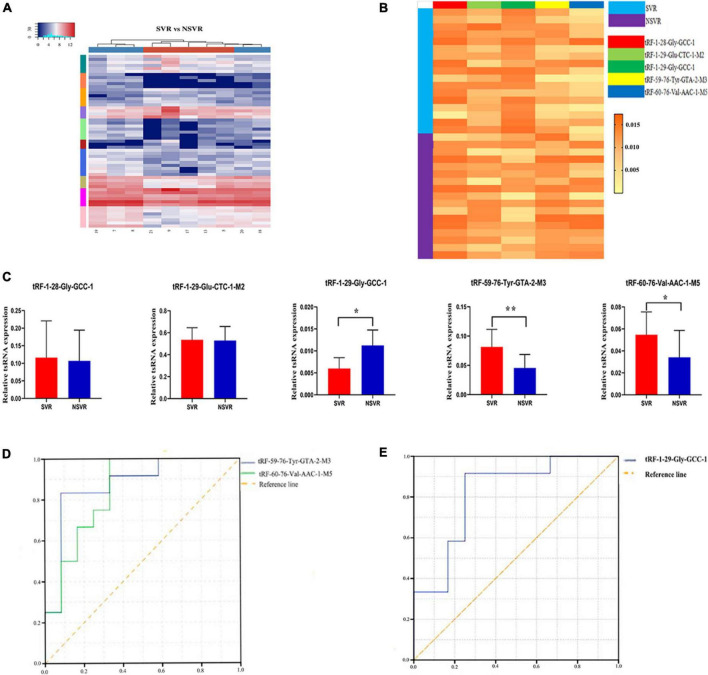
Identification of SVR-related tsRNAs and qPCR confirmation. **(A)** Unsupervised hierarchical clustering heatmap of tRFs and tiRNAs. The color in the panel represents the relative expression level (log_2_-transformed). The color scale is show below: blue represents an expression level below the mean, and red represents an expression level above the mean. The colored bar top at the top panel showed the samples group, and the colored bar at the right side of the panel indicated the divisions which were performed using *K*-means. **(B)** The heatmap of five treatment-related tsRNAs in 17 SVR and 17 NSVR (tRF-1:28-Gly-GCC-1, tRF-1:29-Glu-CTC-1-M2, tRF-1:29-Gly-GCC-1, tRF-59:76-Tyr-GTA-2-M3, and tRF-60:76-Val-AAC-1-M5). The color in the panel represents the relative expression level (log_2_-transformed). The color scale is show below: yellow represents an expression level below the mean, and orange represents an expression level above the mean. **(C)** Real-time PCR verification results of five treatment-related tsRNAs in 17 SVR and 17 NSVR. *P-value < 0.05, **P-value < 0.01. **(D)** ROC curve of upregulated tsRNAs. **(E)** ROC curve of downregulated tsRNAs.

We used a heatmap to cluster the data. The heatmap shows the differential expression of tsRNAs between SVR and NSVR patients (Figure 2A). The upregulated and downregulated tsRNAs are listed in Supplementary Data 1.

We selected five tsRNAs with high FCs and complete data in expression profiles (tRF-1:28-Gly-GCC-1, tRF-1:29-Glu-CTC-1-M2, tRF-1:29-Gly-GCC-1, tRF-59:76-Tyr-GTA-2-M3, and tRF-60:76-Val-AAC-1-M5) to validate the tsRNA-Seq results in 17 SVRs and 17 NSVRs. As shown in Figures 2A–C, with the exception of tRF-1:28-Gly-GCC-1 and tRF-1:29-Glu-CTC-1-M2, the other three differentially expressed tsRNAs were consistent with the results of RNA sequencing. Relative to the NSVR group, the expression of tRF-59:76-Tyr-GTA-2-M3 (*P* < 0.01) and tRF-60:76-Val-AAC-1-M5 (*P* < 0.05) was upregulated, while the expression of tRF-1:29-Gly-GCC-1 (*P* < 0.05) was downregulated in the group with SVR.

We further evaluated the diagnostic value of three differentially expressed tsRNAs using ROC curves. Figure 2D shows the ROC curves of tRF-59:76-Tyr-GTA-2-M3 and tRF-60:76-Val-AAC-1-M5. The areas under the ROC curves were, respectively, 0.875 and 0.847 and were thus greater than 0.5. Figure 2E shows the ROC curve of tRF-1:29-Gly-GCC-1, with downregulated expression, and the area under the ROC curve was 0.819. All of these results indicated that these three tsRNAs were potential biomarkers of the therapeutic heterogeneity of sacubitril/valsartan.

### Revealing the transfer RNA-derived small RNAs-targeted pathway of sacubitril/valsartan resistance by biological function analysis

We used tRFTar (see text footnote 4) and tsRFun (see text footnote 5) to forecast the target mRNAs of the above differentially expressed tsRNAs. The biological function analysis showed that tRF-59:76-Tyr-GTA-2-M3 could target 261 genes, tRF-60:76-Val-AAC-1-M5 could target 517 genes, and tRF-1:29-Gly-GCC-1 could target 336 target genes (Supplementary Data 2).

**FIGURE 3:**
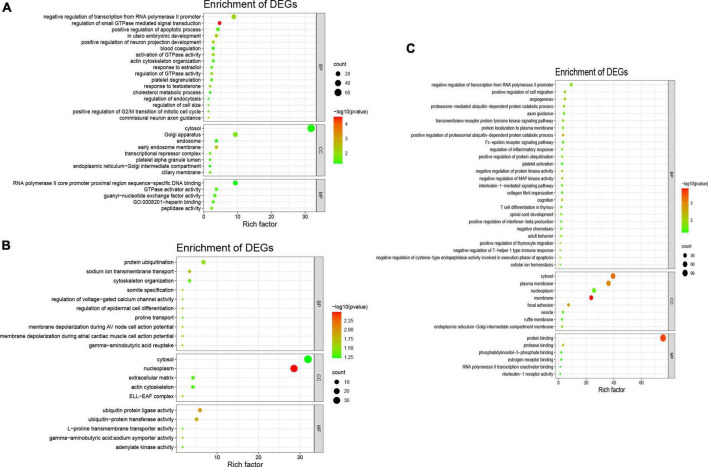
Gene Ontology analysis of three differentially expressed tsRNAs. The GO analysis were composed of biological process (BP), cellular component (CC), and molecular function (MF). **(A)** tRF-59:76-Tyr-GTA-2-M3, the BP were consisted of protein ubiquitination, sodium ion transmembrane transport, cytoskeleton organization, regulation of voltage-gated calcium channel activity, regulation of epidermal cell differentiation, proline transport, membrane depolarization during AV node cell action potential, membrane depolarization during atrial cardiac muscle cell action potential; the CC were consisted of nucleoplasm, cytosol, extracellular matrix, actin cytoskeleton, ELL-EAF complex; and the MF were consisted of ubiquitin protein ligase activity, ubiquitin protein transferase activity, L-proline transmembrane transporter activity. **(B)** tRF-60:76-Val-AAC-1-M5, he BP were consisted of negative regulation of transcription from RNA polymerase II promoter, regulation of small GTPase mediated signal transduction, *in utero* embryonic development, positive regulation of neuron projection development, activation of GTPase activity, regulation of cell size; the CC were consisted of cytosol, Golgi apparatus, early endosome membrane, endosome; and the MF were consisted of RNA polymerase II core promoter proximal region sequence-specific DNA binding, GTPase activator activity, guanyl-nucleotide exchange factor activity, heparin binding, peptidase activity. **(C)** tRF-1:29-Gly-GCC-1, the BP were consisted of angiogenesis, positive regulation of cell migration, negative regulation of transcription from RNA polymerase II promoter, positive regulation of proteasomal ubiquitin-dependent protein catabolic process, negative regulation of MAP kinase activity; the CC were consisted of membrane, cytosol, plasma membrane, focal adhesion; and the MF were consisted of protein binding, protease binding, RNA polymerase II transcription coactivator binding, estrogen receptor binding.

Meanwhile, the results of the GO enrichment analysis for tRF-59:76-Tyr-GTA-2-M3, tRF-60:76-Val-AAC-1-M5, and tRF-1:29-Gly-GCC-1 are shown in Figures 3A–C.

**FIGURE 4:**
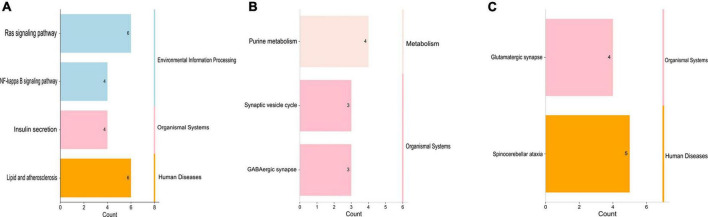
Kyoto Encyclopedia of Genes and Genomes analysis of three differentially expressed tsRNAs. **(A)** tRF-59:76-Tyr-GTA-2-M3, **(B)** tRF-60:76-Val-AAC-1-M5, **(C)** tRF-1:29-Gly-GCC-1. The target gene of tRF-59:76-Tyr-GTA-2-M3 is involved in regulation of the pathways in purine metabolism (PDE6H, AK2, ADCY1, and AK9), synaptic vesicle (SLC6A11, SLC6A1, and AP2A2), and GABAergic synapse (SLC6A11, ADCY1, and SLC6A1); the target gene of tRF-60:76-Val-AAC-1-M5 is involved in regulation of the pathways in lipid and atherosclerosis (CAMK2B, VAV3, PLCB3, NFATC3, TNFRSF10B, and BCL2L1), Ras signaling pathway (PLA2G2F, ANGPT4, RAB5B, RASAL1, BCL2L1, and VEGFA), NF-κB signaling pathway (CCL4, CFLAR, ERC1, and BCL2L1), and Insulin secretion (CAMK2B, PLCB3, KCNJ11, and ATP1B2); while the target gene of tRF-1:29-Gly-GCC-1 was participated in the pathways of spinocerebellar ataxia (XBP1, ITPR2, WIPI2, GRIA3, and ATG2B), and glutamatergic synapse (GRM5, SLC1A2, ITPR2, and GRIA3).

Then, KEGG enrichment analysis was performed, and the results are shown in Figures 4A–C, which indicated that the target genes of tRF-60:76-Val-AAC-1-M5 might participate in regulation *via* the lipid and atherosclerosis pathway, the Ras signaling pathway, the NF-κB signaling pathway and insulin secretion (Table 5).

**TABLE 5:** Kyoto Encyclopedia of Genes and Genomes pathways of the target genes.

tsRNA	Term	*P*-value	Genes
tRF-59:76-Tyr-GTA-2-M3	hsa00230: purine metabolism	0.03	PDE6H, AK2, ADCY1, AK9
	hsa04721: synaptic vesicle cycle	0.07	SLC6A11, SLC6A1, AP2A2
	hsa04727: GABAergic synapse	0.09	SLC6A11, ADCY1, SLC6A1
tRF-60:76-Val-AAC-1-M5	hsa04911: insulin secretion	0.06	CAMK2B, PLCB3, KCNJ11, ATP1B2
	hsa05417: lipid and atherosclerosis	0.07	CAMK2B, VAV3, PLCB3, NFATC3, TNFRSF10B, BCL2L1
	hsa04014: Ras signaling pathway	0.09	PLA2G2F, ANGPT4, RAB5B, RASAL1, BCL2L1, VEGFA
	hsa04064: NF-κB signaling pathway	0.09	CCL4, CFLAR, ERC1, BCL2L1
tRF-1:29-Gly-GCC-1	hsa05017: spinocerebellar ataxia	0.04	XBP1, ITPR2, WIPI2, GRIA3, ATG2B
	hsa04724: glutamatergic synapse	0.08	GRM5, SLC1A2, ITPR2, GRIA3

### Alteration of tRF-60:76-Val-AAC-1-M5-targeted genes enriched in the lipid and atherosclerosis signal pathway after transfection

**FIGURE 5:**
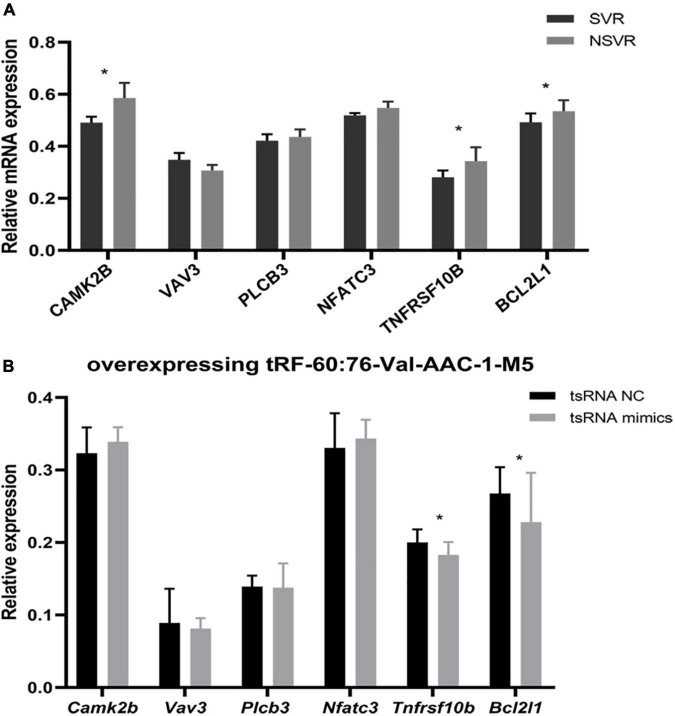
tRF-60:76-Val-AAC-1-M5-targeted genes enriched in lipid signal pathways and atherosclerosis after transfection. **(A)** Differences in mRNA expression between SVR and NSVR patients. **(B)** Relative mRNA levels after the transfection of HUVECs with tRF-60:76-Val-AAC-1-M5. Data are presented as the mean ± SD (*n* = 3 each group), *P < 0.05 indicate significant differences relative to the group of tsRNA NC.

The hsa05417 (lipid and atherosclerosis) pathway is closely related to human diseases. A total of 6 genes were enriched in the lipid and atherosclerosis signaling pathway (CAMK2B, VAV3, PLCB3, NFATC3, TNFRSF10B, and BCL2L1), and these genes were regulated by tRF-60:76-Val-AAC-1-M5. We first compared the relative mRNA expression levels between SVR and NSVR patients and found that the mRNA expression of CAMK2B, TNFRSF10B, and BCL2L1 was lower in the SVR group (Figure 5A).

Next, we carried out an experiment to reveal the relationship between tsRNAs and the target genes of hsa05417 *in vitro*. After overexpressing tRF-60:76-Val-AAC-1-M5 in HUVECs, *Tnfrsf10b* and *Bcl2l1* were remarkably downregulated (*P* < 0.05), but *Camk2b, Vav3, Plcb3*, and *Nfatc3* showed no change (*P* > 0.05) (Figure 5B). These results indicated that tRF-60:76-Val-AAC-1-M5 might target *Tnfrsf10b* and *Bcl2l1* to influence the therapeutic heterogeneity of sacubitril/valsartan through the lipid and atherosclerosis signaling pathway.

## Discussion

Sacubitril/valsartan, which was the first dual enkephalinase and angiotensin receptor inhibitor to be developed, is a monocrystal formed by sacubitril and valsartan at a molar ratio of 1:1 (5). Sacubitril can be transformed into the active compound LBQ657 through metabolic processes and can inhibit the degradation of endogenous natriuretic peptides by enkephalinase, resulting in reduced sympathetic tension and relaxed blood vessels and promoting the excretion of urine and sodium (27). The other component, valsartan, can selectively act on the angiotensin II type I receptor, thereby reducing the production and release of angiotensin and aldosterone and inhibiting heart injury caused by excessive RAAS activity (28). Hence, this drug plays a dual cardioprotective role in AMI patients with HF. However, many AMI patients with HF do not benefit to cardiac function after treatment with sacubitril/valsartan. Results obtained from the CHAMP-HF registry (29) revealed that after treatment with sacubitril/valsartan, LVEF declined in 19% of patients and remained unchanged in 31%. These findings may be related to sex, drug doses, baseline LVEF, etiology, pacemaker implantation, and other factors, but the specific mechanism is not clear.

Transfer RNA-derived small RNAs are a type of non-coding RNA with regulatory functions formed by the precise cleavage of tRNAs, including tRFs and tiRNAs, which can exert biological activity in a variety of ways (30). An increasing number of researches have reported that tsRNAs are inextricably linked with the pathological process of various diseases, especially tumors, inflammatory and immune diseases. In the cytoplasm of patients with breast cancer, mutated nucleotides have been identified in the tRNA-His-GUG at the 5′ end of the 5′ half. ANG selectively cleaves the tRNA missing the −1 nucleotide to form this specific tsRNA, which then accumulates, thus accelerating the progression of breast cancer (31). Another study showed that after Rickettsia bacteria infect the human body, they carry exogenous ANG into the nucleus of endothelial cells, activate RNA transcription, and then quickly enter the cytoplasm to induce the production of the smaller tRF-5S to promote the phosphorylation reaction of endothelial adhesion protein and reduce its stability, thereby weakening the function of the endothelial barrier and leading to the development of a maculopapular rash (32). Therefore, tsRNA has become a hotspot in various medical researches these days.

In addition to the participation of tsRNA in the regulation of the occurrence and development of many disorders, for instance, tumors, alcoholic fatty liver, neurodegenerative diseases, infectious diseases, and stress diseases, tsRNA has now been confirmed to produce a marked effect on cardiovascular diseases (21, 22). tsRNA is also closely related to ventricular remodeling. Myocardial hypertrophy induced by isoproterenol was identified in an animal experiment. The expression of tsRNA in a rat model was shown to be more abundant than that in a healthy control group, in which tRFs1 and tRFs2 were positively correlated with the expression of the cardiac hypertrophy factors ANF, β-MHC, and NBP (33). Another study also showed that tRF-5 was abundant in the sperm of mice with myocardial hypertrophy and inhibited the mRNA expression of the hypertrophy regulator TIMP3 by binding its 3′UTR sequence, thereby aggravating cardiomyocyte hypertrophy, increasing cardiac fibrosis and promoting apoptosis (34).

During this investigation, since we evaluated the expression profiles of tsRNAs and compared the differential expression of tsRNAs between SVR and NSVR patients and found that relative to the NSVR group, the expression of tRF-59:76-Tyr-GTA-2-M3 and tRF-60:76-Val-AAC-1-M5 was upregulated, while the expression of tRF-1:29-Gly-GCC-1 was downregulated in the group with SVR. ROC curve analysis revealed that these three tsRNAs were potential biomarkers of the therapeutic heterogeneity of sacubitril/valsartan. Another pharmacological study of Buyang-Huanwu decoction also indicated that tsRNA could be a novel therapeutic target in intracerebral hemorrhage (35). Although a study with a larger sample size will help further verify our conclusion, this study is the first to address the genetic contribution to the heterogeneity of sacubitril/valsartan and has revealed some key tsRNAs. All of these findings provide new perspectives for future exploration to explain the underlying mechanisms.

Moreover, we performed GO enrichment analysis of the mRNAs targeted by tsRNAs and showed that the upregulated tsRNA-targeted mRNAs were mainly involved in the regulation of calcium and sodium transmembrane transport and action potential depolarization in atrial myocytes and atrioventricular node cells, among other BPs. Due to ischemia or the necrosis or apoptosis of cardiomyocytes in patients with AMI, ventricular contractility is reduced and heart pumping function is weakened, and this condition is often complicated by HF. The electrical conduction function of cardiomyocytes is destroyed simultaneously, which causes the heart to contract asynchronously, further increasing the development of stress failure. The upregulated differentially expressed tsRNAs may regulate the contractile activity of cardiomyocytes by affecting ion channels. The downregulated tsRNA-targeted mRNAs were mainly involved in angiogenesis. It is important to increase cardiac perfusion and promote the establishment of collateral circulation after myocardial infarction to improve the prognosis. A continuous cardiac hypoperfusion state leads to a progressive decline in cardiac function and even cardiogenic shock. Downregulated differentially expressed tsRNAs may significantly decrease cardiac function by inhibiting vascular remodeling and collateral angiogenesis.

When we carried out enrichment analysis of KEGG pathway, we discovered that the target mRNAs of tRF-60:76-Val-AAC-1-M5 were enriched in the lipid and atherosclerosis signaling pathway, NF-κB signaling pathway, and Ras pathway, which are involved in the regulation of SVR. Previous articles have reported that the above three pathways are intensively correlated to the inflammatory immune response, atherosclerosis and angiogenesis (36, 37). Although tRNA was one of the highly modified RNAs in cells, a latest study found that unmodified tRF-3 could also result in gene silencing through luciferase reporting experiments (38). In this study, our group used unmodified tRF fragments for overexpression research and confirmed that tRF-60:76-Val-AAC-1-M5 might target Tnfrsf10b and Bcl2l1 to influence the therapeutic heterogeneity of sacubitril/valsartan through the lipid and atherosclerosis signaling pathway. In AMI patients with HF, if a lipid metabolism disorder or arteriosclerosis progress cannot be controlled, it is not conducive to the regeneration of blood vessels, the recovery of the myocardium and the improvement of cardiac function after myocardial infarction. Second, NF-κB is the most important regulator of the inflammatory response (39). Studies have shown that under the stress imposed by AMI (40), proinflammatory factors bind to TNF receptors on the cell membrane and activate the specific phosphorylation of the serine residue of κB, resulting in the activation of NF-κB, its entry into the nucleus; in the nucleus, NF-κB acts on gene promoters to accelerate the pathological process of atherosclerosis by advancing an inflammatory response, damaging the vascular endothelium, promoting the proliferation and migration of VSMCs and promoting vascular cell apoptosis (41). Finally, studies have revealed that RAS is closely related to angiogenesis (42). VEGF is a specific mitogen of endothelial cells that targets endothelial cells and plays a large role in promoting endothelial cell mitosis and angiogenesis. Activated Ras can activate the VEGF transcription factor through the Ras-Raf-ERK1/2 signaling pathway (43), and RAS can improve the stability of VEGF mRNA through the Ras-Rac-MEKK1-JNKK signaling pathway (44) to enhance the expression of VEGF and promote angiogenesis (45). All of the above signaling pathways might participate in the regulation of cardiac function after MI and provide new insights for investigating SVR mechanisms.

Hence, on the basis of the present study, it could construct a new prediction system for identifying the patients promptly without a clear benefit from sacubitril/valsartan in HF patients after AMI, and it could significantly help to select patients benefiting from alternative approaches such as cardiac resynchronization therapy (CRT), device therapy, and even LVAD implantation. What was more, the above findings would give the opportunity to better classify the molecules and pathways activated in SVR patients, which might represent an additional benefit to identifying the new molecular targets to improve the outcome of the optimal medical therapy. Although we made great efforts to avoid errors in this research, this study still has inherent limitations. First, the drug response might be affected by additional factors, such as pharmacodynamics and the internal environment. The possible influence of these confounding factors on our results cannot be excluded. Moreover, is was a single-center study, in which the population included were the patients with AMI complicated by HF. The confirmation of the obtained conclusion will require a larger sample size to increase the reliability of present results. Finally, *in vivo* animal validation studies and a more in-depth mechanistic exploration will improve our understanding in the future.

## Conclusion

To sum up, this research determined the expression profiles of tsRNAs in SVR and NSVR patients and found that the expression of tRF-59:76-Tyr-GTA-2-M3 and tRF-60:76-Val-AAC-1-M5 was upregulated, while the expression of tRF-1:29-Gly-GCC-1 was downregulated in the group with SVR. ROC curve analysis indicated that these three tsRNAs can potentially be used as biomarkers of the therapeutic heterogeneity of sacubitril/valsartan. Moreover, tRF-60:76-Val-AAC-1-M5 might target Tnfrsf10b and Bcl2l1 to influence this therapeutic heterogeneity through the lipid and atherosclerosis signaling pathway. These discoveries provide new insights for the mechanistic investigation of the responsiveness of sacubitril/valsartan and will help clinicians identify prognostic benefits in patients to realize individualized treatment.

## Data availability statement

Publicly available datasets were analyzed in this study. These data can be found here: https://www.ncbi.nlm.nih.gov/geo/query/acc.cgi?acc=GSE207882.

## Ethics statement

The studies involving human participants were reviewed and approved by the Ethics Committee of Ningbo No. 1 Hospital. The patients/participants provided their written informed consent to participate in this study. Written informed consent was obtained from the individual(s) for the publication of any potentially identifiable images or data included in this article.

## Author contributions

JS, KZ, WD, and XC designed the experiments. JC, YH, QY, ZL, JL, ZeZ, XL, and JS carried out the experiments. NZ, ZhZ, and JY analyzed the experimental results. JS, JC, and YH wrote the manuscript. YW, WD, and XC contributed to helping with project administration and manuscript review and editing. All authors contributed to the article and approved the submitted version.

## Footnotes

1
http://gtrnadb.ucsc.edu/

2
http://lowelab.ucsc.edu/tRNAscan-SE/

3
https://www.ncbi.nlm.nih.gov/geo/

4
http://www.rnanut.net/tRFTar/

5
https://rna.sysu.edu.cn/tsRFun/

6
https://www.ncbi.nlm.nih.gov/geo/query/acc.cgi?acc=GSE207882

## Supplementary material

Supplementary Data 1 The upregulated and downregulated tsRNAs in SVR.

Supplementary Data 2 The target mRNAs of three differentially expressed tsRNAs.
