# Supplementary material for: Transfer RNA-derived small RNAs and their potential roles in the therapeutic heterogeneity of sacubitril/valsartan in heart failure patients after acute myocardial infarction
Source: Front Cardiovasc Med. 2022 Sep 29;9:961700. doi: 10.3389/fcvm.2022.961700 (PMC9558900; doi:10.3389/fcvm.2022.961700)
Supplement: Supplementary file 1 [file Table_1.docx]

TableS1 Quality score

| Sample | Total Read | TotalBase | Base（Q≥30） | Base Q≥30）（%） |
| --- | --- | --- | --- | --- |
| T1 | 6114212 | 305710600 | 290913372 | 95.16 |
| T2 | 7718127 | 385906350 | 366485238 | 94.97 |
| T3 | 7019755 | 350987750 | 333895380 | 95.13 |
| T4 | 7842972 | 392148600 | 373320044 | 95.20 |
| T5 | 6385199 | 319259950 | 302646186 | 94.80 |
| C1 | 8166730 | 408336500 | 389637142 | 95.42 |
| C2 | 7526041 | 376302050 | 358422055 | 95.25 |
| C3 | 10984846 | 549242300 | 521999755 | 95.04 |
| C4 | 7544827 | 377241350 | 357634366 | 94.80 |
| C5 | 7555569 | 377778450 | 359796010 | 95.24 |

Table S2 Mapping summary for reads statistical information.

| Sample | Trimmed | Mature tRNA | Mature tRNA(%) | Precursor tRNA | Precursor tRNA(%) | miRNA | miRNA(%) |
| --- | --- | --- | --- | --- | --- | --- | --- |
| C1 | 5931161 | 157493 | 2.66 | 30956 | 0.52 | 2990827 | 50.43 |
| C2 | 6378235 | 167777 | 2.63 | 26557 | 0.42 | 3097783 | 48.57 |
| C3 | 8879064 | 244299 | 2.75 | 71997 | 0.81 | 4794997 | 54.00 |
| C4 | 6096436 | 173098 | 2.84 | 49391 | 0.81 | 3125887 | 51.27 |
| C5 | 6548659 | 154539 | 2.36 | 35297 | 0.54 | 3228455 | 49.30 |
| T1 | 4825526 | 112503 | 2.33 | 39023 | 0.81 | 2612098 | 54.13 |
| T2 | 6382737 | 190504 | 2.98 | 28976 | 0.45 | 2378869 | 37.27 |
| T3 | 5396467 | 124822 | 2.31 | 35138 | 0.65 | 2849930 | 52.81 |
| T4 | 5732607 | 131355 | 2.29 | 35209 | 0.61 | 2161455 | 37.70 |
| T5 | 4306994 | 98183 | 2.28 | 17082 | 0.40 | 1372409 | 31.86 |
